# A High-Resolution SNP Array-Based Linkage Map Anchors a New Domestic Cat Draft Genome Assembly and Provides Detailed Patterns of Recombination

**DOI:** 10.1534/g3.116.028746

**Published:** 2016-03-29

**Authors:** Gang Li, LaDeana W. Hillier, Robert A. Grahn, Aleksey V. Zimin, Victor A. David, Marilyn Menotti-Raymond, Rondo Middleton, Steven Hannah, Sher Hendrickson, Alex Makunin, Stephen J. O’Brien, Pat Minx, Richard K. Wilson, Leslie A. Lyons, Wesley C. Warren, William J. Murphy

**Affiliations:** * Department of Veterinary Integrative Biosciences, Interdisciplinary Program in Genetics, Texas A&M University, College Station, Texas 77843; † McDonnell Genome Institute, Washington University School of Medicine, St. Louis, Missouri 63108; ‡ College of Veterinary Medicine, University of Missouri-Columbia, Missouri 65201; § Population Health and Reproduction, University of California-Davis, California 95616; ** Institute for Physical Sciences and Technology, University of Maryland, College Park, Maryland 20742; †† National Cancer Institute-Frederick, National Institutes of Health, Maryland 21702; ‡‡ Nestlé Purina PetCare Company, St. Louis, Missouri 63134; §§ Department of Biology, Shepherd University, Shepherdstown, West Virginia 25443; *** Theodosius Dobzhansky Center for Genome Bioinformatics, St. Petersburg State University, Russia

**Keywords:** *Felis catus*, Illumina 63K SNP array, genetic map, recombination, domestic cat

## Abstract

High-resolution genetic and physical maps are invaluable tools for building accurate genome assemblies, and interpreting results of genome-wide association studies (GWAS). Previous genetic and physical maps anchored good quality draft assemblies of the domestic cat genome, enabling the discovery of numerous genes underlying hereditary disease and phenotypes of interest to the biomedical science and breeding communities. However, these maps lacked sufficient marker density to order thousands of shorter scaffolds in earlier assemblies, which instead relied heavily on comparative mapping with related species. A high-resolution map would aid in validating and ordering chromosome scaffolds from existing and new genome assemblies. Here, we describe a high-resolution genetic linkage map of the domestic cat genome based on genotyping 453 domestic cats from several multi-generational pedigrees on the Illumina 63K SNP array. The final maps include 58,055 SNP markers placed relative to 6637 markers with unique positions, distributed across all autosomes and the X chromosome. Our final sex-averaged maps span a total autosomal length of 4464 cM, the longest described linkage map for any mammal, confirming length estimates from a previous microsatellite-based map. The linkage map was used to order and orient the scaffolds from a substantially more contiguous domestic cat genome assembly (*Felis catus* v8.0), which incorporated ∼20 × coverage of Illumina fragment reads. The new genome assembly shows substantial improvements in contiguity, with a nearly fourfold increase in N50 scaffold size to 18 Mb. We use this map to report probable structural errors in previous maps and assemblies, and to describe features of the recombination landscape, including a massive (∼50 Mb) recombination desert (of virtually zero recombination) on the X chromosome that parallels a similar desert on the porcine X chromosome in both size and physical location.

During the past decade, increasingly detailed gene maps and sequence assemblies of the domestic cat genome have provided tools for mapping and discerning the heritable basis of morphological variation and genetic diseases (Murphy *et al.* 2007; Pontius *et al.* 2007; Bach *et al.* 20012; Davis *et al.* 2009; Menotti-Raymond *et al.* 2009; Mullikin *et al.* 2010; Montague *et al.* 2014). These include mutations in functional genes that control coat morphology (*e.g.*, Lyons *et al.* 2005; Schmidt-Küntzel *et al.* 2005; Ishida *et al.* 2006; Gandolfi *et al.* 2013a,b; Montague *et al.* 2014; Lyons 2015), as well as those that are causative for monogenic diseases such as hypertrophic cardiomyopathy (Meurs *et al.* 2005), progressive retinal atrophy in different breeds (Menotti-Raymond *et al.* 2007; Alhaddad *et al.* 2014), and many others (see Gandolfi and Alhaddad 2015; Lyons 2015 for current reviews). The ability to narrow candidate regions and identify causative mutations in the feline model is currently limited by the quality of the domestic cat genome assembly and an inadequate genetic variant database.

The recent development of a moderate-density (∼63,000 marker) Illumina SNP array has transitioned the mapping of feline traits from family-based linkage approaches to GWAS for simple and polygenic traits (Gandolfi *et al.* 2012, 2013a,b; Alhaddad *et al.* 2014; Davis *et al.* 2015). Here, we demonstrate the utility of the 63K SNP array for generating a high-density genetic linkage map based on genotyping data from several large multigenerational domestic cat pedigrees. High-density linkage maps have been produced for a number of mammals, including human (Kong *et al.* 2002), mouse (Shifman *et al.* 2006; Cox *et al.* 2009), cattle (Arias *et al.* 2009), pig (Tortereau *et al.* 2012), and dog (Wong *et al.* 2010). These resources provide valuable recombination information that complement genome sequence data, allowing for an improved understanding of the landscape of genetic linkage in mapping and association studies.

Prior feline genetic maps have been constructed from genotyping microsatellite markers in both interspecies backcross pedigrees (Menotti-Raymond *et al.* 1999, 2003) and domestic cat multigenerational pedigrees (Grahn *et al.* 2005; Menotti-Raymond *et al.* 2009; Schmidt-Küntzel *et al.* 2005). The average marker density of the latter map was relatively low (483 microsatellite markers, ∼1 marker/9 Mb), and possessed some discrepancies with feline radiation hybrid maps (Menotti-Raymond *et al.* 2009; Davis *et al.* 2009). Nonetheless, the existing resources have provided crucial information for the initial genetic mapping of most feline phenotypic traits and diseases. Here, we describe the production of high-density sex-averaged recombination maps for each domestic cat chromosome based on genotyping data from the Illumina 63K feline SNP array. These high-resolution maps were then used in the generation of an improved whole genome assembly that was not heavily reliant on human–dog conserved gene order for scaffolding. The new assembly incorporated 20 × coverage of Illumina reads into the data used for the previous version (v6.2, Montague *et al.* 2014) genome assembly (felCat5). The new maps and assembly correct a number of structural errors in the previous assembly, and allow for the first detailed examination of recombination rate patterns across the domestic cat genome.

## Materials and Methods

### Pedigrees and genotyping

Three independent pedigrees of domestic cats were used in the calculation of the recombination maps. The first is a five-generation extended pedigree of cats maintained by Nestlé Purina Company, consisting of 309 nonbreed cats (Menotti-Raymond *et al.* 1999), 305 of which were successfully genotyped on the array. The second pedigree had founders of Persian, Oriental Shorthair, Bengal, Japanese Bobtail, American Curl, and Somali that were crossbred to maximize diversity while retaining diseases and traits of interest. The colony was bred from 1999–2013, producing a nine-generation crossbreed pedigree. We included 165 genotyped cats from this second colony in our analysis. Genomic DNA was extracted from whole blood or tissue using either standard phenol chloroform extraction or the DNeasy Blood and Tissue Kit (Qiagen). DNA from all cats were genotyped on the Illumina feline 63K SNP BeadChip. Only SNPs with call rates > 0.95 and no replication errors were included in the construction of the final maps. Samples with < 0.90 call rate were removed from the analysis. Following Mendelian inheritance checks, 453 final cats were evaluated in the final linkage analysis.

### Linkage analysis

We used the software package CRI-MAP (Green *et al.* 1990) v2.504 to build linkage maps for each chromosome using the combined Illumina 63K SNP genotype data derived from each of the 453 domestic cats. We removed 2365 markers that were included in the array design that assay felid lineage-specific apomorphies and phenotype-associated mutations, were uninformative, or possessed poor genotyping quality across samples. The *two-point* and *autogroup* options were used to cluster SNP markers into linkage groups based on pairwise LOD scores among all informative markers, enforcing a LOD score threshold of 8.0 to ensure no spurious cross-chromosome instances of linkage. The chromosomal assignments for each SNP marker were cross-checked and validated by BLAST (Altschul *et al.* 1990) comparison to the v6.2 genome assembly (Davis *et al.* 2015; Li *et al.* 2016).

Given the large number of markers assayed on the SNP array for each chromosome, we constructed an initial framework map that was then used to successively position more markers at less stringent thresholds of marker inclusion and number of informative meiosis (IM). The *build* option of CRI-MAP was used to construct a framework map for each chromosome/linkage group using only markers with > 300 IM and a LOD threshold of 3.0. Enforcing this LOD 3.0 framework marker order, we then used a sliding window-based approach to position additional markers from each chromosome relative to the framework marker order, because it was computationally prohibitive to position all remaining markers across an entire chromosome in a single analysis. Using the marker coordinates from the v6.2 assembly, we selected 500 markers in consecutive windows moving from the p terminus to the q terminus of each chromosome, for positioning within the LOD-3 chromosome framework maps. Successive windows on each chromosome contained 100 overlapping markers to ensure contiguity of marker order. For each window, markers were added relative to the framework map using successively less-stringent LOD thresholds of 2.0, 1.0, and 0.5. The minimum IM for the latter stages was 100. Markers that could not be positioned at LOD > 0.5 were positioned into their most likely marker order (minimum LOD = 0.1) but were not given a position in the final map. We used the *flips* option (6 marker windows per calculation) to evaluate and refine local marker ordering at each step of the process. We also iteratively examined maps for putative “expander” markers (those that inflated maps > 3 cM), identified by larger-than-average flanking marker intervals. Local maps were rebuilt following removal of each expander marker, and map distances recalculated and compared with the previous map. In the final stage, we placed all remaining candidate markers relative to their most likely marker interval. The final maps and source data may be found in Supplemental Material, File S1 and Table S1.

### Construction of the v8.0 genome assembly

To construct a new genome assembly we utilized all existing genomic data generated from Cinnamon, a female Abyssinian cat used for all prior genome assemblies (Pontius *et al.* 2007; Montague *et al.* 2014), with the exception of felCat4 (Mullikin *et al.* 2010), which also included reads from multiple breeds and a wild cat (*Felis silvestris lybica*). The data from prior maps included ∼2 × whole genome coverage of Sanger-based sequencing (6.7 million plasmid and 1.3 million 40 kb fosmid end reads; Pontius *et al.* 2007), ∼12 × whole genome coverage with 454 sequencing (6 × fragment and 6 × of 3 kb paired-end reads), and Sanger-based end-sequenced BACs (Amplicon Express *Felis catus* FSCC library) (Montague *et al.* 2014). Here, we generated ∼20 × coverage of nonoverlapping 100 bp paired-end reads from a single Illumina short-insert (avg. length = 350 bp) library, prepared from Cinnamon’s DNA, on the HiSeq2000 (SRA Accession numbers SRX478589 and SRX478590). The new Illumina reads were combined with all prior sequence reads from Cinnamon (described above) to generate a new *de novo* assembly (v7.0) using the MaSuRCA assembler version 1.9.2 (Zimin *et al.* 2013). To ensure minimal loss of prior assembly sequence data, we merged the v6.2 assembly with the new v7.0 MaSuRCA assembly to create the final assembly, v8, using GAA (Yao *et al.* 2012), deposited under accession number GCA_000181335.3. A final processing step was deployed to fill small (< 1 kb) within-scaffold gaps using custom scripts to align and assemble Illumina reads.

To build individual chromosomes, we used BLASTN (E-value of 1e-9, with percent identity threshold = 85%) to align flanking probe sequences for the Illumina SNP markers (used in the linkage map construction) to the v8.0 genome assembly scaffolds. In addition, the assembled cat genome was broken into 1 kb segments and aligned against the v6.2 domestic cat assembly, the dog genome (canFam3), and the human genome (hg19) using BLAT (Kent 2002) with identity threshold = 85%, to precisely identify uniquely aligning segments of the cat genome that aided in identifying syntenic breakpoints down to very fine resolution. The new v8.0 genome scaffolds were ordered and oriented along 1) the v6.2 cat chromosomes, to check for larger scale scaffolding errors and or alignment inconsistencies, and 2) the new linkage map to validate, and in some cases modify, their order and orientation along the entire chromosome.

BLASTZ and BLAT alignments of the v8.0 assembly to the dog and human genomes were then used to refine the order and orientation information, as well as to insert additional scaffolds that may not have been tagged by markers on the linkage map. These new scaffolds were placed into the conditional scaffold framework provided by the initial v6.2/v8.0 cat alignments and marker assignments. Alignments between the cat, dog, and human genomes were used to more precisely identify the breakpoints within scaffolds when marker and alignment information confirmed a false join within the genome assembly. In addition, 98 finished BAC clone sequences (primarily selected from the FCAB library made from Cinnamon, with 8 MHC-spanning clones from the RPCI-86 library) were also incorporated into the genome assembly. Satellite sequences were identified in the v8.0 assembly, and centromeres were placed along each chromosome using the localization data from the radiation hybrid (RH) map of Davis *et al.* (2009).

### Data availability

The authors state that all data necessary for confirming the conclusions presented in the article are represented fully within the article.

## Results

### Recombination map

We removed 35 markers with Mendelian inheritance violations, and 2478 markers with fewer than 100 IM during the marker ordering step. The final maps included 58,055 markers, including 8402 unique map positions at LOD ≥ 0.5 that were ordered relative to an initial framework map of 2204 LOD > 3.0 markers distributed across the 18 autosomes and X chromosome. 6659 of the 8402 markers were positioned at LOD > 1.0 and 4978 at LOD > 2.0. The final map provided an ordered and positioned density of ∼24 markers/Mb (Table 1).

**Table 1:** Comparison of previous domestic cat radiation hybrid and linkage maps with the new SNP array-based linkage map (see Table S1)

Chromosome	Radiation Hybrid Map (Davis *et al.* 2009)				Microsatellite Genetic Linkage Map (Menotti-Raymond *et al.* 1999; Schmidt-Küntzel *et al.* 2005)			63K SNP Array Genetic Linkage Map (Present Study)			
	v8.0 Physical Length (Mb)	Markers	Unique Map Positions	Marker/Mb*^a^*	Markers	Map Length (cM)	cM/Mb*^a^*	Total Markers/Framework Markers	Total Markers/Mb	Map Length (cM)	cM/Mb*^b^*
A1	246	246	129	1.0	40	337.5	1.4	6219/217	25.3	414.6	1.7
A2	180	205	127	0.9	32	235.7	1.1	4529/208	25.2	315.8	1.8
A3	143	155	97	0.9	26	230.2	1.5	3162/175	22.1	275.5	1.9
B1	198	190	104	1.0	48	367.2	1.9	4636/210	23.4	305.4	1.5
B2	148	140	90	1.1	25	218.8	1.6	3612/186	24.4	267.2	1.8
B3	143	144	96	1.0	20	218.8	1.5	2934/172	20.5	278.0	1.9
B4	138	148	88	0.9	27	293.5	2.0	3633/194	26.3	276.0	2.0
C1	220	197	125	1.1	41	430.2	2.2	5677/208	25.8	424.1	1.9
C2	148	158	98	0.9	28	230.4	1.5	3726/175	25.2	244.0	1.6
D1	123	133	83	0.9	31	228.8	1.7	3129/172	25.4	231.5	1.9
D2	103	105	58	1.0	27	221.4	2.1	2279/167	22.1	208.5	2.0
D3	103	114	80	0.9	19	146.4	1.3	2685/144	26.1	199.6	1.9
D4	95	109	69	0.9	18	167.2	1.5	2334/172	24.6	216.5	2.3
E1	95	109	62	0.9	18	194.4	1.8	1659/128	17.5	204.9	2.2
E2	78	82	52	0.9	21	186.9	2.3	1736/130	22.3	159.6	2.0
E3	60	69	50	0.9	9	112.4	1.6	1259/85	21.0	126.9	2.1
F1	75	102	56	0.7	17	150.3	1.5	1865/125	24.9	158.3	2.1
F2	75	84	47	0.9	34	172.4	2.1	2248/156	30.0	157.1	2.1
X	128	172	91	0.7	46	228.4	1.3	733/82	5.7	161.0/109*^c^*	1.3/0.85*^c^*
Total/average	2493	2662	1602	0.9	527	4370.9	1.6	58,055/2204	24.0*^d^*	4624.5	1.9

SNP, single nucleotide polymorphism.

a Based on comparison to the felCat3 (1.9X) genome assembly.

b Based on comparison to the v8.0 genome assembly.

c Excluding the pseudoautosomal region.

d Autosomes only.

The sex-averaged length of the autosomal map was 4464 cM, just 2% longer than the 4370 cM map length based on microsatellite genotyping (Menotti-Raymond *et al.* 2009). Calculating map length from only 2204 LOD > 3.0 framework markers also resulted in a similarly long map estimate of 4048 cM, suggesting our results were not heavily biased by inclusion of more than 6000 additional markers positioned at lower LOD thresholds (Table 1). The overall map lengths and chromosome-wide recombination rates were very similar to previous estimates based on comparison to a lower quality genome assembly (Menotti-Raymond *et al.* 2009). Female autosomal map length (5857 cM) was 1.7 × longer than the male map length (3415 cM), consistent with expectations from previous feline linkage maps (Menotti-Raymond *et al.* 2009).

The sex-averaged recombination rate across the genome was 1.9 cM/Mb, with rates varying from a high of 2.3 cM/Mb on chromosome D4, to a low of 0.85 cM/Mb on the X chromosome outside of the pseudoautosomal region (PAR) (Table 1). Recombination rates were highest near telomeric and subtelomeric regions when compared to centromeres, which typically contained the lowest recombination rates for each biarmed chromosome (Figure 2). Similar patterns of increased recombination near chromosome ends have been reported in other mammals (*e.g.*, Paigen *et al.* 2008; Wong *et al.* 2010). However, this pattern was not as apparent on the acrocentric cat chromosomes F1 and F2 (Figure 3).

The cat X chromosome possessed a large recombination coldspot that spans ∼50 Mb, including the centromere. Notably, this same coldspot was “painted” in FISH mapping experiments that hybridized multiple, individual BAC clones selected from within this general region of the X chromosome to feline metaphase spreads (Davis *et al.* 2009). In contrast, FISH results from more than 100 individual BAC clones from outside of the X coldspot and on other autosomes, produced only discrete FISH signals on individual autosomes or distal portions of Xp and Xq, but not a paint-like FISH hybridization signal (Davis *et al.* 2009) (Figure 3). We therefore interpret this painting effect to be due to the presence of an unidentified repetitive gene family or enrichment of a class of repetitive elements within these X-specific BAC clones, which are unique to or highly enriched within the central portion of the X chromosome.

### A new domestic cat genome assembly

The new v8.0 genome assembly incorporates ∼2.56 Gb of sequence (excluding Ns in gaps) that were ordered and oriented on the 18 autosomes and X chromosome (Table 2). An additional 146 Mb of scaffolds have probable chromosome assignments (chr*_random), and ∼76 Mb of unplaced scaffolds have no known assignment (chr_UN). The size of the UN is likely an underestimate as these scaffolds are generally short, repetitive sequences that have been collapsed and have high sequence read depth. In total, this constitutes a 12% increase in assembly length, although the total gap length remained nearly the same (41 *vs.* 40 Mb) because more sequence was incorporated into the assembly. The v8.0 assembly contig and scaffold N50 lengths were 45 kb and 18 Mb, respectively, an increase of nearly 2.1 × and 3.8 × over the v6.2 assembly (Table 2).

**Table 2:** Domestic cat v8.0 genome assembly statistics compared to v6.2

Assembly Version	Assembled Size	N50 Contig Size	N50 Scaffold Size	Total Gap Size	Reference
*F. catus* v6.2	2.36 Gb	21 kb	4.7 Mb	40 Mb	Montague *et al.* 2014
*F. catus* v8.0	2.64 Gb	45 kb	18 Mb	41 Mb	Present study

**Figure 1:**
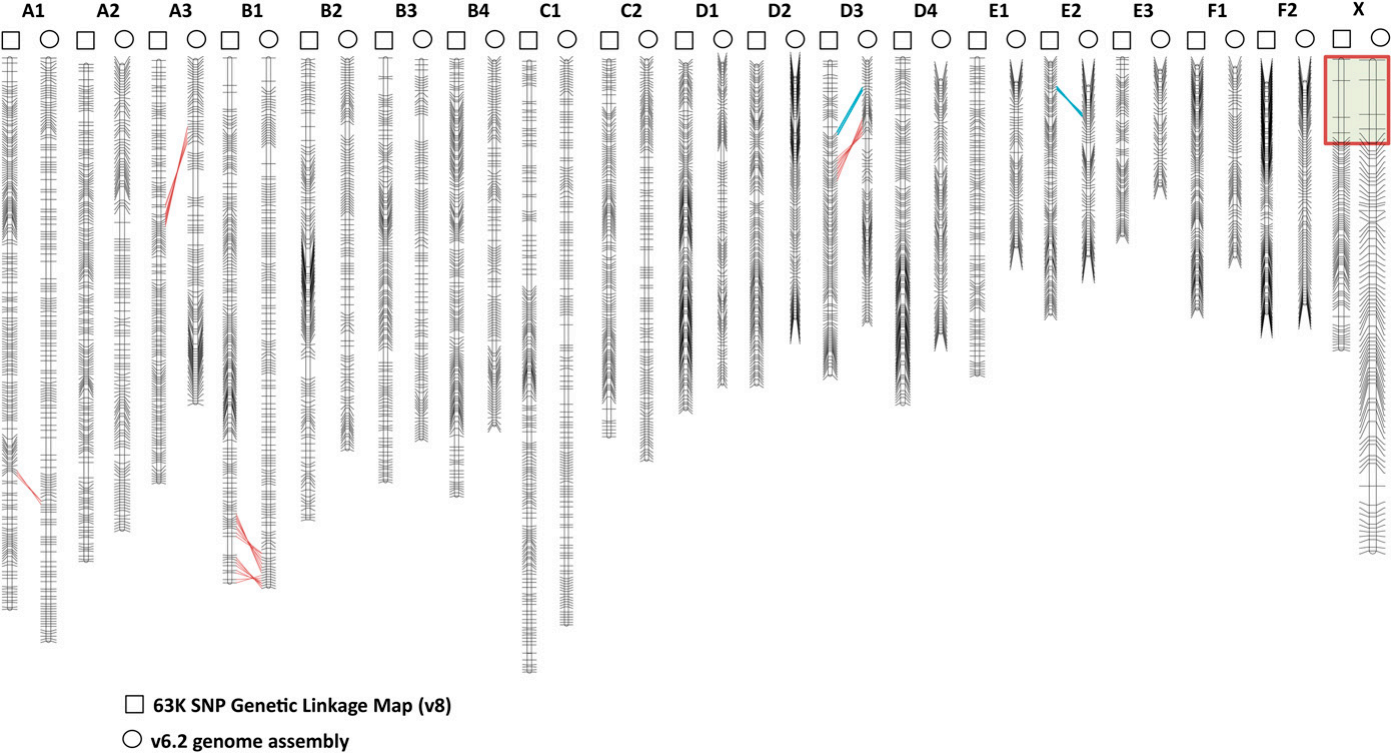
Domestic cat SNP linkage map (indicated above by squares) comparison with the v6.2 genome assembly (indicated above by circles). Black horizontal lines indicate framework marker (LOD 3.0) positions on both maps. Discordant marker orders are displayed between the maps with red (inversions) and blue (translocations) lines. The red box on the X chromosome maps indicate the pseudoautosomal region markers. LOD, logarithm of the odds; SNP, single nucleotide polymorphism.

We examined the integrity of the v8/linkage map chromosome marker orders by aligning the SNP-based linkage map with published RH and microsatellite-based linkage maps, which were previously used to guide the assembly of the domestic genome sequence (v6.2) (Table 1). The new high-density linkage map confirmed the larger scale order of markers in the v6.2 assembly (Montague *et al.* 2014) but also identified several structural differences with the v6.2 assembly (Figure 1). These include five inversions (chr. A1, chr. A3, two on chr. B1, and chr. D3) and two translocations (chr. D3 and chr. E2).

## Discussion

Production of “draft” quality mammalian genome assemblies has grown tremendously over the last few years with reduced costs of next-generation sequence data production. Despite the increase in throughput, these initial “blueprints” are often insufficient for detailed genetic investigations of the molecular signatures of selection or disease-causing alleles of particularly complex traits (Alkan *et al.* 2011; Zhang *et al.* 2009; Kirby *et al.* 2013). Given the substantial progress to date in feline trait mapping, coupled with the availability of genomic resources, there is continued motivation to improve the quality of the cat reference genome to further facilitate mapping of simple and complex traits of biomedical, aesthetic, and evolutionary interest.

We constructed a high-resolution genetic linkage map of the domestic cat genome, based upon Illumina 63K SNP genotypes from 453 domestic cats in three multi-generational pedigrees. The 63K array was designed with 62,897 SNPs ascertained from six domestic cats and an African wild cat (*F. lybica*) (Mullikin *et al.* 2010). The average marker spacing on the array is ∼1 SNP marker every 50 kb. The new SNP-based linkage map provides a > 20-fold increase in improvement with regard to marker density compared to previous linkage (Menotti-Raymond *et al.* 2009; Schmidt-Küntzel *et al.* 2005) and RH (Murphy *et al.* 2007; Davis *et al.* 2009) maps. In addition, the contiguity and accuracy of the current reference genome sequence has improved markedly over previous assembly versions used to estimate chromosome wide recombination rates (Menotti-Raymond *et al.* 2009), hence facilitating the resolution of finer-scale domestic cat recombination rates across the genome.

The new reference genome presented here incorporates 280 Mb of new sequence data into the assembly, and shows a substantial improvement of contiguity as a result of new sequence data and use of the high-resolution linkage map. Compared to v6.2, the v8.0 assembly showed 2.2 and 3.9-fold increases in contig and scaffold N50 lengths, respectively, and corrected ∼7 larger misassemblies that may have impacted GWAS interpretation. These discrepancies may have resulted from parts of the v6.2 assembly that relied heavily on published lower-density RH and linkage maps (Figure 1). Furthermore, a majority of scaffolds in the v6.2 assembly were ordered and oriented based on extrapolations from conserved human–dog gene order because they were not tagged by a genetic marker from the existing lower resolution linkage and RH maps.

### Recombination landscape

The detailed linkage map allowed for the first precise estimates of recombination rates across the genome. At 4464 cM, the domestic cat possesses the longest sex-averaged autosomal recombination map length of any eutherian mammal studied to date. This result is corroborated by independent cytogenetic estimates of meiotic rates of recombination in felids, which showed domestic cat and tiger possess the highest rates of any other analyzed eutherian mammals (Segura *et al.* 2013). Our new map length is also consistent with an independent and equally long estimate of 4370 cM observed by Menotti-Raymond *et al.* (2009), based on genotyping of 483 autosomal microsatellites on a smaller cohort of domestic cats. In addition to these maps, an earlier, less contiguous genetic linkage map calculated from genotyping 328 microsatellite loci in an interspecies hybrid backcross hybrid pedigree between the domestic cat and the Asian leopard cat (*Prionailurus bengalensis*) produced a map of 3300 cM, also relatively longer than linkage maps of most other eutherian species (Menotti-Raymond *et al.* 2003). The genome-wide sex-averaged recombination rate for the autosomes was 1.9 cM/Mb, also higher than all other reported mammalian genetic maps, and two to three times the rate of genetic maps for pig (0.85 cM/Mb, Tortereau *et al.* 2012), dog (0.97 cM/Mb, Wong *et al.* 2010), and mouse (0.63 cM/Mb, Shifman *et al.* 2006).

**Figure 2:**
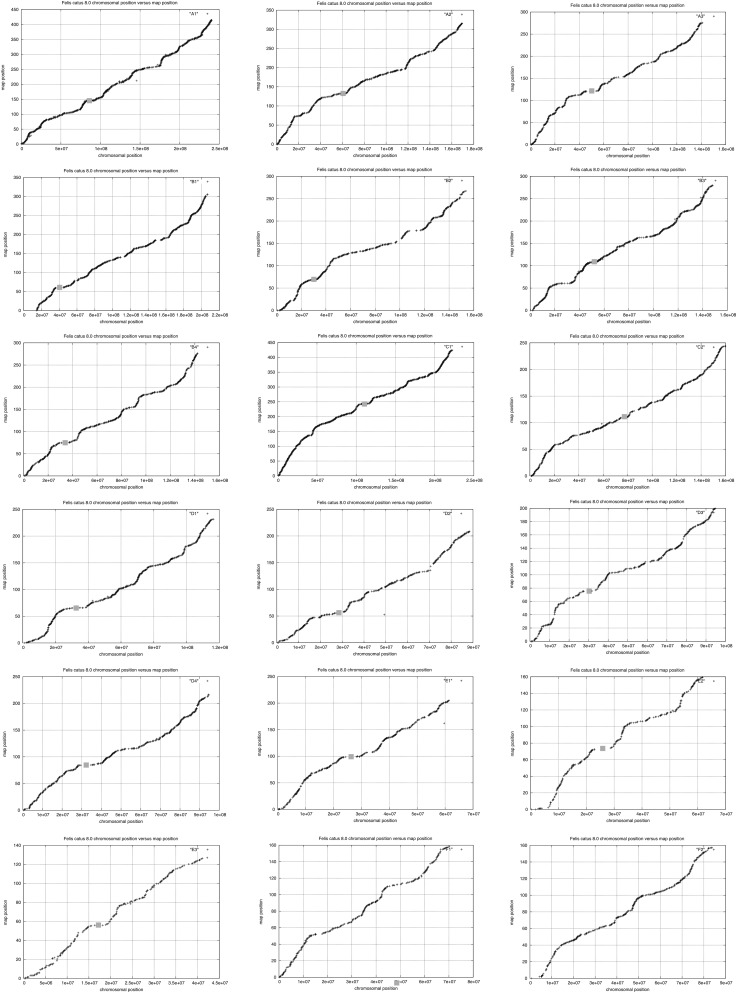
Plots of recombination distance (*y* axis, in cM) *vs.* physical distance (*x* axis, v8.0 assembly, in bp) for each of the 18 feline autosomes (identified in the upper right corner of each plot). Centromeres are indicated by a gray box.

**Figure 3:**
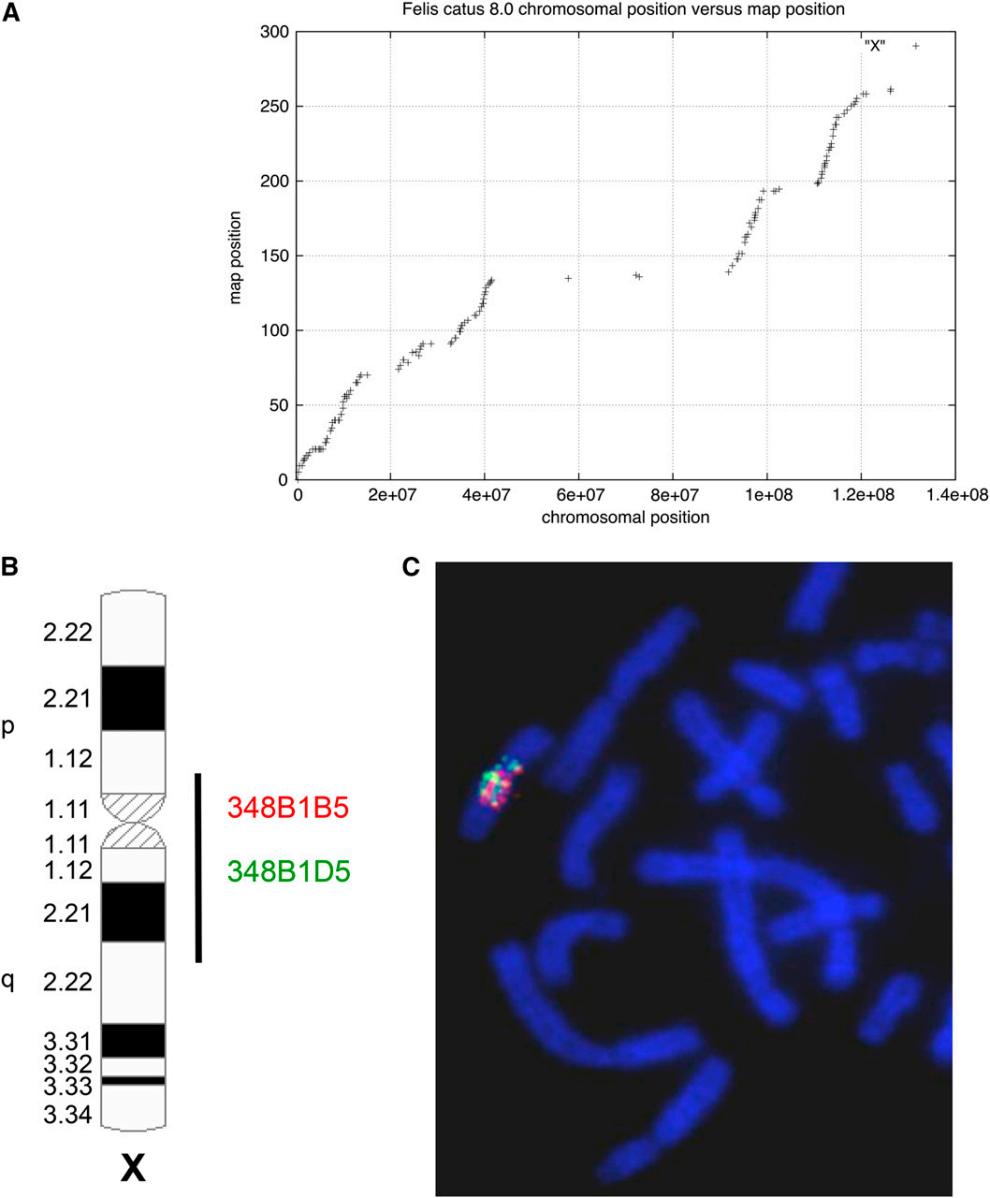
(A) Plot of recombination distance (*y* axis, in cM) *vs.* physical distance (*x* axis, v8.0 assembly, in bp) for the X chromosome. (B) Ideogram showing the distribution of hybridization signals of two randomly selected domestic cat BAC (bacterial artificial chromosome) clones (from Davis *et al.* 2009) derived from the centrally located X chromosome recombination desert. (C) Both clones paint the entire chromosomal region corresponding with the approximate boundaries of the recombination desert.

The distribution of recombination rates was quite variable along each chromosome. Recombination rates were reduced around most, but not all, centromeres, and increased in subtelomeric/telomeric regions (Figure 2). The most notable feature is an extremely long (∼50 Mb) recombination desert (with virtually no recombination) on the center of chromosome X (Figure 3), in addition to two smaller recombination coldspots on Xp and distal Xq. These cold regions are flanked by recombination hotspots. A similarly large recombination coldspot, also flanked by high recombination rate regions, was described from the domestic pig X chromosome in a colinear region of conserved synteny (Ma *et al.* 2010; Ai *et al.* 2015). Furthermore, this region in cat and pig is colinear with the human X chromosome (Murphy *et al.* 1999; Davis *et al.* 2009; Ma *et al.* 2010); a smaller but overlapping region surrounding the human X centromere also shows reduced recombination (Weeks *et al.* 1995).

Recently, Ai *et al.* (2015) and Li *et al.* (2016) observed similar signatures of interspecies genetic introgression within the large X chromosome recombination coldspot between high and low-altitude populations of pigs, and big cats of the genus *Panthera*, respectively. Both sets of authors posited that positive selection was a driving force behind the introgression patterns enriched on this region of the X chromosome, as natural selection is most effective in low-recombination regions (Smith and Haigh 1974). Selection is particularly effective on the X chromosome, where both beneficial and deleterious recessive alleles are exposed in hemizygous males (Charlesworth *et al.* 1987). Ai *et al.* (2015) reported enrichment of a thymidine-rich repeat within this region, and similarly, Davis *et al.* (2009) noted that BAC clones isolated from this region of the X chromosome also produced paint-like FISH signals on domestic cat chromosome spreads, implying a conserved X chromosome landmark across eutherian mammal orders (Figure 3).

Previous studies have hypothesized that selection during domestication events could potentially result in elevated recombination rates (Burt and Bell 1987; Ross-Ibarra 2004; Coop and Przeworski 2007), although recent studies have questioned this association (Muñoz-Fuentes *et al.* 2015). Indeed, in their analysis of selection signatures associated with cat domestication, Montague *et al.* (2014) observed a significant enrichment in genes involved in homologous recombination (*e.g.*, *BRCA2*, *RAD51B*, and *ZFYVE26*) within regions of high differentiation between wild and domestic cats. The authors also identified a putative case of adaptive linkage (*sensu*, Otto and Barton 1997) between two regulators of homologous recombination (*RAD51B* and *ZFYVE26*) and a candidate domestication gene (*PLEKHH1*). Considering these facts, together with the evidence presented here that the domestic cat recombination rate is higher than any other studied eutherian mammal, our data lend support to the hypothesis that the process of domestication may have driven an increase in the domestic cat’s rate of recombination. Future analyses of chiasma frequencies in wild cat predecessors of the domestic cat would test this hypothesis.

### Future prospects

The domestic cat genome assembly is unique among many other mammalian genome assemblies, being based on a large variety of sequencing read lengths, including a combination of legacy and NGS technology, and is anchored by a variety of physical mapping resources, including 2 × Sanger sequence data, multiple genetic linkage and RH maps, and fosmid and BAC-end sequences. This combination of mapping technologies has resulted in an extremely well-validated chromosome level assembly. These improvements to the assembly, coupled with whole genome SNP arrays, have led to a surge in trait and disease mapping efforts in cats, similar to advances in canine trait and disease mapping following the release of a high coverage draft assembly (Karlsson and Lindblad-Toh 2008).

The majority of the v8.0 draft genome sequence coverage is, nonetheless, primarily based on short NGS reads (454 and Illumina), which are known to inhibit the construction of highly contiguous *de novo* genome assemblies (Alkan *et al.* 2011; Bradnam *et al.* 2013, Sudmant *et al.* 2015, Chaisson *et al.* 2015a,b). As a result, we estimated that approximately 10–15% of the genome sequence (∼480 million bp) remains in gaps or cannot be assigned to unique chromosomal positions at this time due to mapping ambiguity arising from repetitive structures, particularly segmental duplications. These regions are therefore not accessible to GWAS using the 63K array.

A growing body of evidence suggests that redundant genomic elements (*i.e.*, duplications) are important contributors to health-related phenotypes, possibly due to overlapping, interchangeable, or even novel tissue-specific functions of slightly differentiated, physically separated sequences that regulate gene expression (Zhang *et al.* 2009; Girirajan *et al.* 2012; Sudmant *et al.* 2010, 2015). Many phenotypes that are of interest to cat breeders, such as disease and disease-resistance, pattern and coloration, and reproduction, are regulated by genes that are members of gene families that are segmentally duplicated and copy number variable (Li *et al.* 2013, Montague *et al.* 2014). It is well known that these rapidly evolving, dynamic regions are often collapsed or even absent from draft genome assemblies (Church *et al.* 2009; Alkan *et al.* 2011; Chaisson *et al.* 2015a). These observations imply that a significant amount of feline phenotypic variation is likely to be modulated by genes or gene families imbedded within repetitive structures (*e.g.*, David *et al.* 2014, Davis *et al.* 2015). While the current genome assembly is a major step forward toward generating a more complete reference, the future incorporation of data from long single molecule sequencing (*e.g.*, Pacific Biosciences, 10 × Genomics) and other emerging scaffolding technologies (*e.g.*, BioNano Genomics and Dovetail Genomics maps) will lead to additional improvements in the quality and completeness of the domestic cat genome (English *et al.* 2012; Chaisson *et al.* 2015b, Steinberg *et al.* 2014).

## Supplementary Material

Supplemental Material
