# Supplementary material for: A High-Resolution SNP Array-Based Linkage Map Anchors a New Domestic Cat Draft Genome Assembly and Provides Detailed Patterns of Recombination
Source: G3 (Bethesda). 2016 Mar 29;6(6):1607–16. doi: 10.1534/g3.116.028746 (PMC4889657; doi:10.1534/g3.116.028746)
Supplement: Supplemental Material [file supp_6_6_1607__index.html]

A High-Resolution SNP Array-Based Linkage Map Anchors a New Domestic Cat Draft Genome Assembly and Provides Detailed Patterns of Recombination — Supplemental Material 

# A High-Resolution SNP Array-Based Linkage Map Anchors a New Domestic Cat Draft Genome Assembly and Provides Detailed Patterns of Recombination

## Supplemental Material for Li *et al.*, 2016

**Files in this Data Supplement:**

- Table S1 - Final linkage maps. (.xlsx, 2 MB)
- File S1 - Pedigree and genotype information. (.txt, 70 MB)
